# Public knowledge and preventive behavior during a large-scale Salmonella outbreak: results from an online survey in the Netherlands

**DOI:** 10.1186/1471-2458-14-100

**Published:** 2014-01-31

**Authors:** Lex van Velsen, Desirée JMA Beaujean, Julia EWC van Gemert-Pijnen, Jim E van Steenbergen, Aura Timen

**Affiliations:** 1 National Institute of Public Health and the Environment, Centre for Infectious Disease Control, P.O. box 1, 3720 BA Bilthoven, The Netherlands; 2 Department of Psychology, Health and Technology, University of Twente, P.O. box 217, 7500AE Enschede, The Netherlands; 3 Leiden University Medical Center, P.O. Box 9600, 2300 RC Leiden, The Netherlands

**Keywords:** Food-borne infections, Gastrointestinal disease, Hygiene, Infection control, Outbreaks, Public health policy, *Salmonella*

## Abstract

**Background:**

Food-borne *Salmonella* infections are a worldwide concern. During a large-scale outbreak, it is important that the public follows preventive advice. To increase compliance, insight in how the public gathers its knowledge and which factors determine whether or not an individual complies with preventive advice is crucial.

**Methods:**

In 2012, contaminated salmon caused a large *Salmonella Thompson* outbreak in the Netherlands. During the outbreak, we conducted an online survey (n = 1,057) to assess the general public’s perceptions, knowledge, preventive behavior and sources of information.

**Results:**

Respondents perceived *Salmonella* infections and the 2012 outbreak as severe (m = 4.21; five-point scale with 5 as severe). Their knowledge regarding common food sources, the incubation period and regular treatment of *Salmonella* (gastro-enteritis) was relatively low (e.g., only 28.7% knew that *Salmonella* is not normally treated with antibiotics). Preventive behavior differed widely, and the majority (64.7%) did not check for contaminated salmon at home. Most information about the outbreak was gathered through traditional media and news and newspaper websites. This was mostly determined by time spent on the medium. Social media played a marginal role. Wikipedia seemed a potentially important source of information.

**Conclusions:**

To persuade the public to take preventive actions, public health organizations should deliver their message primarily through mass media. Wikipedia seems a promising instrument for educating the public about food-borne *Salmonella*.

## Background

With an estimated 80.3 million cases each year, food-borne *Salmonella* infections are a worldwide concern [1]. In developing areas in Africa, Asia and South-America, *Salmonella Typhi* and *Paratyphi* are an important cause of severe illness, leading to more than 20 million cases and 200.000 deaths in children and young people every year [2]. A typical *Salmonella* infection can lead to fever, diarrhea, nausea, vomiting, abdominal cramps, and headache. Symptoms usually appear between 24 to 48 hours after eating contaminated food, and last three to seven days. The incidence rate of *Salmonella* is highest among infants and young children. As there are many different types of food-borne *Salmonella*, each with their own food sources, control is difficult. Proper hygiene in the kitchen (e.g., washing hands, thoroughly heating and baking meat) can prevent a *Salmonella* infection. However, studies among the general public in Italy [3], Turkey [4] and New Zealand [5] showed that compliance with preventive hygiene advice is low to very low. A possible explanation is that most people believe that a food-borne infection is “something that happens to others” [6,7].

Educating the public about food safety is crucial in preventing food-borne infections. According to Medeiros and colleagues [8], food-borne *Salmonella* infections should be prevented by educating the general public about adequate cooking of food, and by instructing them about the risks of cross-contamination. Traditional communication means, such as flyers, are well suited to achieve these educational goals [9]. However, when a food-borne infection breaks out on a large scale, the dynamics of the situation shift tremendously. Due to an uncertain course of events, decisions have large consequences, the general public is stressed, and the media is eager for news [10]. In these circumstances, health organizations should inform the public about the situation and persuade them to take preventive actions. To be effective in this endeavor, they should use the communication channels the general public expects them to use, and provide the public with the information they want and need. A study among Malaysians during the A(H1N1) influenza outbreak in 2009, uncovered that their main sources of information were newspapers, television and family members; their information needs were instructions on how to prevent or treat infections [11]. In the Netherlands, the 2003 *Severe Acute Respiratory Syndrome* (SARS) outbreak and the 2011 *Enterohaemorrhagic E. Coli* (EHEC) outbreak showed us that the Dutch general public mostly turns to traditional media (i.e., television and radio), and news websites [12,13].

In recent years, the rise of social media (e.g., Facebook, Twitter) has provided new avenues for reaching the general public during infectious disease outbreaks. Although social media have proven very valuable during disaster relief as a crowdsourcing tool [14], an exploratory study of their worth as a communication tool during an infectious disease outbreak suggested their value to be limited [13]. Research on the information behavior of the general public during infectious disease outbreaks is scarce. But this knowledge is crucial in serving the general public in their information needs, and in maximizing citizen compliance with preventive advice.

In this study, we uncovered the general public’s perceptions, knowledge, preventive behavior, and sources of information during a large, national *Salmonella* outbreak by a large-scale online survey. As a result, we were able to answer our main research question: Which information should health organizations convey during a large-scale *Salmonella* outbreak, and by which channels, to maximize citizen compliance with preventive advice?

## Methods

### Case

In the beginning of August 2012, an outbreak of *Salmonella Thompson* occurred in the Netherlands [15], later traced back to contaminated smoked salmon from one producer. By September 28, all smoked salmon of this producer was recalled. In the following week, other products containing this producer’s smoked salmon (e.g., salads) were also recalled. Citizens were advised to check the batch number of their products and to dispose of possible contaminated products. After implementing those measures, the number of cases decreased rapidly and by the end of 2012, the outbreak came to an end. 1,149 laboratory-confirmed patients and four deaths were reported [16]. The actual number of patients is thought to be higher, as individual cases of *Salmonella* gastro-enteritis are not mandatory notifiable in the Netherlands and laboratory confirmation usually merely takes place in a fraction of all patients presenting with diarrhea. According to Dutch standards, this situation classifies as a large-scale outbreak, as it is an occurrence of disease greater than would otherwise be expected at a particular time and place. Normally around four cases of *Salmonella Thompson* are seen in the Netherlands per year.

### Survey

We developed an online survey to assess the general public’s perceptions, knowledge, preventive behavior, and information use during the 2012 *Salmonella Thompson* outbreak. The instrument was constructed on the basis of the Health Belief Model [17], and research on citizen channel choice for medical information [18,19]. The survey contained 35 questions, and was divided into five domains:

1. Perceptions, in which we assessed perceived severity of a *Salmonella* infection and the 2012 outbreak, interest in health information, and perceived health.

2. Knowledge about *Salmonella* infections.

3. Preventive behavior, where we questioned the presence of a *Salmonella* infection in the participant’s vicinity, their application of measures to prevent a *Salmonella* infection during the outbreak (by eating less salmon, being careful with buying salmon, and by buying canned instead of fresh salmon), and increased general kitchen hygiene during the outbreak.

4. Sources of information, in which we assessed participants’ information intake about the outbreak through the media, and where they went to look for answers to questions related to *Salmonella* infections and the outbreak.

5. Demographics.

Perceptions were assessed by multiple statements with five-point Likert scales (ranging from disagree (1) to agree (5)). Items were based on Bults et al. [20]. Knowledge was assessed by nine true/false statements. Preventive behavior was assessed by multiple-choice questions about what respondents did after hearing about the outbreak. Sources of information were determined by questioning how often and where respondents saw, heard or read about the outbreak. Next, we asked respondents if they had wanted more information about the outbreak or an answer to a specific question about the outbreak. If so, we asked where they had sought this information or the answer. If they had so through the Internet, then we asked them if they had found it through a Google search, whether they had found what they were looking for, how satisfied they were with the website, and how much they trusted the information. To keep the length of the survey acceptable, we only posed these questions for one website the participants named. If they named more than one website, the website was chosen at random. The survey can be found in Additional file 1.

Respondents were recruited by a commercial panel that also hosted the survey in their online environment. The panel supplied standard demographics for each respondent (e.g., age and income). A stratified sample was taken to create a representative group of the Dutch population. The minimum age for participation was 18 years. The target sample size was 1,000 respondents, to allow for satisfactory statistical power, and to maximize our chances of including people who contracted a *Salmonella* infection. Respondents received points for participating, with which they could buy gifts in an online shop. Panel participants received an individual invitation via email of which the first was sent out on November 13, 2012. The survey was closed on November 20, 2012. Due to the method of recruitment, a response rate could not be calculated.

Written informed consent was obtained from each respondent for publication of this report. The nature of this general internet-based survey among healthy volunteers from the general population does not require formal medical ethical approval according to Dutch law [21].

### Analyses

Descriptive statistics were performed for the demographics, respondents’ preventive behavior, and sources of information. Cronbach’s alpha was calculated to assess internal consistency for the psychological rating scales. These scores were .85 for perceived severity of *Salmonella,* .84 for perceived severity of the 2012 outbreak, .77 for carefulness with salmon preparation during the outbreak, .89 for carefulness with general food preparation during the outbreak, .80 for interest in health information, and .76 for perceived health. Next, mean scores were computed for the aforementioned psychological rating scales, while the statements for assessing knowledge about *Salmonella* infections resulted in a sum score (ranging from 0 to 9, where 0 is no knowledge and 9 is very high knowledge). To establish the influence of factors determining respondents’ application of preventive measures during the outbreak (dependent variable), we performed stepwise backward regression analyses. Following [22-24], we included the following independent variables in the initial model: the demographics age, education, income and sex, and the factors perceived severity of a *Salmonella* infection, perceived severity of the outbreak, knowledge about *Salmonella* infections, and increased general kitchen hygiene during the outbreak. Education was recoded into a new variable with three options: Low, middle or high, while sex was included in the regression analyses as a dummy variable. These actions make it possible to include these nominal variables in this kind of regression analysis. Factors were removed from the model if *p* > .10. The procedure was repeated for determining the factors that influence the consumption of information about the 2012 *Salmonella* outbreak for different media. Here, consumption of information on a medium was the dependent variable for the different models (each model explaining the information consumption for a specific medium.). We included the following independent variables in the initial models: the demographics age, having children, education, income, and sex (based on [18,19]), as well as the factors perceived severity of a *Salmonella* infection, perceived severity of the *Salmonella* outbreak, knowledge about *Salmonella* infections, interest in health information, and perceived health (based on [18]), as well as the application of measures to prevent a *Salmonella* infection, and increased carefulness with preparing food (following [25]). For the variables using Twitter or not, and having children or not, we also created a dummy variable. These analyses allowed us to formulate recommendations in line with our main research question:

Which information should health organizations convey during a large-scale *Salmonella* outbreak, and by which channels, to maximize citizen compliance with preventive advice?

## Result

### Demographics

**Figure 1:**
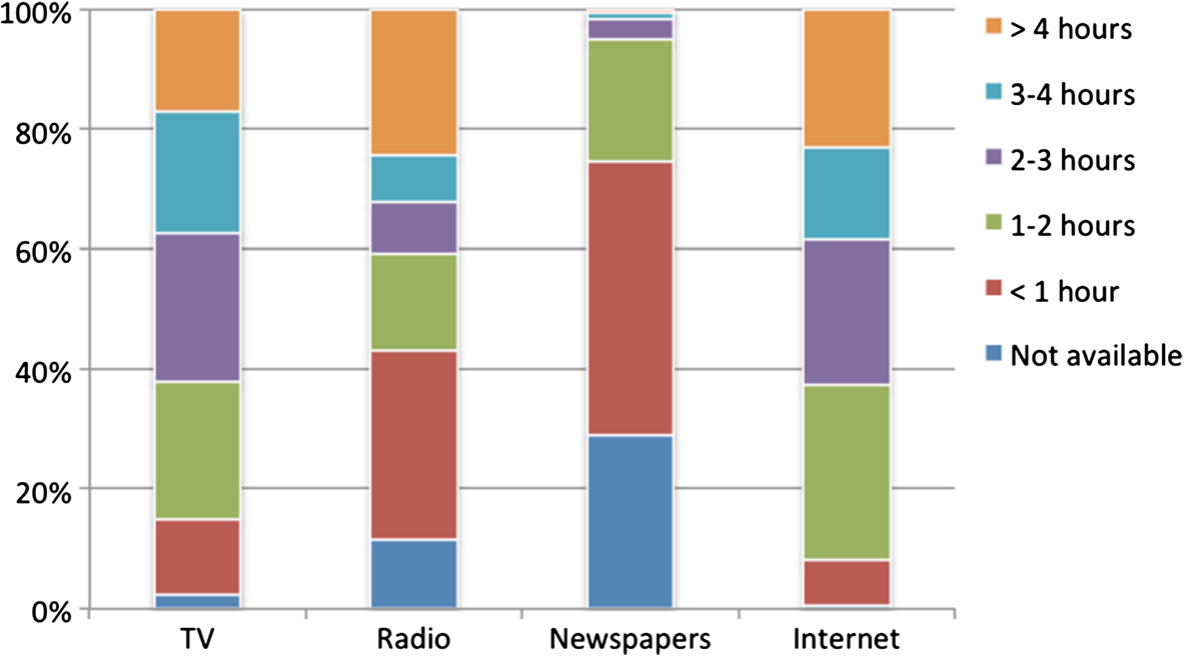
Respondents’ media use (n = 1,057).

In total, 1,057 respondents completed the survey. Table 1 displays their demographics, showing that the sample is fairly representative for the Dutch population. Figure 1 shows how often the respondents made use of different media. Most respondents watched television more than two hours a day. Radio was less popular, although one quarter listened to this medium more than four hours a day. The majority spent some time each day reading a newspaper. Most respondents used the Internet intensively. Finally, 29.6% had a Twitter account, 45.5% a Hyves (a Dutch social network) account, and 73.0% a Facebook account.

**Table 1:** Respondent demographics (n = 1,057)

	**Sample**	**Dutch population**
**Sex**		
Male	49.4%	49.5%
Female	50.6%	50.5%
**Age**		
< 20	1.2%	3.1%
20 to 40	36.4%	31.3%
40 to 65	43.6%	45.1%
65 to 80	16.1%	15.3%
> 80	2.7%	5.2%
**Family situation**		
Single, no children	31.8%	36.8%
Single, with children	5.8%	6.8%
Living together or married; no children	26.1%	29.2%
Living together or married; with children	34.8%	27.3%
Other	1.5%	0.6%
**Education**		
Low	40.8%	31.7%
Intermediate	36.0%	40.5%
High	23.2%	27.9%
**Ethnicity**		
Dutch	84.9%	79.1%
Other	15.1%	20.9%
**Daily occupation**		
School	9.3%	
Entrepreneur	2.7%	
Paid commercial job	31.0%	
Paid non-profit job	19.0%	
Houseman/-wife	9.4%	
Incapacitated	6.4%	
Unemployed	4.4%	
Retired	16.4%	
Other	1.5%	
**Income**		
Minimum	9.4%	
Below average	22.1%	
Average	18.8%	
1-2 times average	19.6%	
> 2 times average	7.9%	
Do not know	5.7%	
Do not want to disclose	16.4%	

Available data for Dutch population from Statistics Netherlands [26]; numbers for education level Dutch population for ages 15–65 years.

### Perceptions

Respondents perceived *Salmonella Thompson* to be quite a severe infection (m = 4.21; SD = .76). This finding is corroborated by the comparison respondents made between a *Salmonella* infection and other illnesses. This comparison is displayed in Table 2, and shows that *Salmonella* is estimated as severe as asthma and diabetes. The 2012 outbreak was also estimated as quite severe (m = 4.12; SD = .86). Respondents’ mean interest in health information (m = 3.15; SD = .71), and their perceived health (m = 3.49; SD = .77) were neutral.

**Table 2:** Respondents’ estimation of severity of illnesses (n = 1,057)

**Illness**	**Mean severity**	**S.D.**
Ordinary flu	2.51	.97
Diabetes	4.34	.80
Heart attack	4.83	.43
*Salmonella* infection	4.13	.89
Asthma	4.23	.84
HIV or AIDS	4.88	.38

### Knowledge

We assessed respondents’ knowledge about *Salmonella* infections by nine true/false statements (see Table 3). The respondents appeared to be well informed, with a few exceptions. 39% was unaware of the common sources of a *Salmonella* infection, 47,5% unaware of its incubation period, and 71,3% was unaware of how *Salmonella* is treated in general. We calculated a sum score for each respondent’s knowledge (with a maximum of 9). The mean score was 6.91 (SD = 1.11).

**Table 3:** **Respondents’ knowledge of *Salmonella *** **infections (n = 1,057)**

	**Correct answer**	**Percentage of correct respondent replies**
People can get *Salmonella* from eating contaminated food.	True	97.4%
*Salmonella* can develop into a very serious infection, especially with babies and the elderly.	True	96.3%
When there is an outbreak (several people are suffering from *Salmonella*), the Municipal Health Service tries to identify the source.	True	96.2%
Most of the time, people get *Salmonella* from others who are already infected.	False	91.3%
By properly washing and heating food, you can prevent getting ill from *Salmonella*.	True	86.8%
If you have symptoms from *Salmonella* (like vomiting or diarrhea), you are temporarily not allowed to work in healthcare.	True	81.2%
*Salmonella* can predominantly be found on chicken, raw vegetables, and fruit.	True	61.0%
After you have eaten *Salmonella-*contaminated food, it can take weeks before you become ill.	False	52.5%
*Salmonella* is almost always treated with antibiotics.	False	28.7%

### Preventive behavior

Respondents’ self-reported application of measures to prevent a *Salmonella* infection during the outbreak was below the neutral point (m = 2.35; SD = 1.07), as was their estimation of an increase in kitchen hygiene during the outbreak (m = 2.31; SD = 1.03). However, in both cases standard deviations are quite high, implying that there were people who increased their kitchen hygiene tremendously, and people who absolutely did not. Our regression analysis showed that the application of preventive measures (dependent variable) was influenced by increased general kitchen hygiene during the outbreak (β = .61; *p* < .001), by perceived severity of the outbreak (β = .19; *p* < .001), and by the demographics income (β = .07; *p* < .01) and sex (higher for women; β = .05; *p* < .05). A significant beta means that a factor influences the dependent variable (in this case application of preventive measures). A low beta stands for a small influence, a high beta for a large influence. In this case, the betas show that four factors influence the application of preventive measures; of which increased general kitchen hygiene is by far the greatest influence. Explained variance (R^2^) for the model was .51 (which means that the dependent variable is explained for a large part by the identified independent variables, but also by some, as of yet, unidentified variables).

In our sample, eight respondents (.8%) indicated to have gotten a *Salmonella* infection from eating contaminated salmon. A larger group (26 respondents; 2.5%) knew someone in their close vicinity (friends or family) who ate contaminated salmon and then got a *Salmonella* infection.

We asked the respondents whether they checked if they had salmon at home when they heard of the outbreak. It turned out that:

•275 respondents (26.0%) checked but did not have salmon at home;

•99 respondents (9.4%) checked and did have salmon at home;

•684 respondents (64.7%) did not check if they had salmon at home.

Next, we assessed what the 99 respondents did who had salmon at home:

•55 respondents (55.2%) found out their salmon was not contaminated;

•22 respondents (22.6%) threw all salmon away;

•3 respondents (3.2%) found out they had contaminated salmon and threw it away;

•6 respondents (6.5%) found out they had contaminated salmon, but did eat it;

•12 respondents (12.5%) did something else, mostly returning contaminated salmon to the supermarket.

### Sources of information

In assessing the information behavior of the general public during the *Salmonella* outbreak, we made a distinction between *passive* and *active* information behavior [27]. Passive information behavior consists of situations in which a person receives information without actively searching for it (e.g., listening to the radio, stumbling upon an item when surfing on a news website). In other words, a person is exposed to information without a direct and specific need for this information. Active information is caused by a question or explicit need for information, after which a person actively seeks out information.

**Figure 2:**
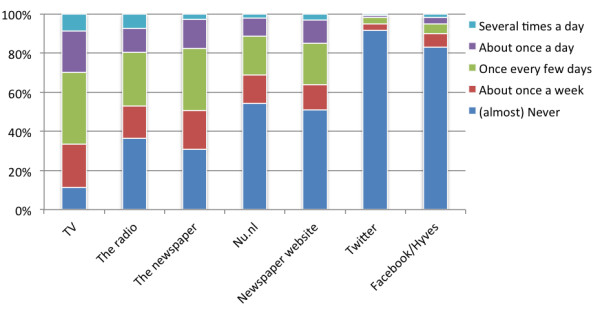
**Number of times news about the *Salmonella *outbreak was received per source (n = 1,057).** Note: nu.nl is a popular news website in the Netherlands.

Figure 2 displays the channels and popular online sources from which the respondents have passively received information about the *Salmonella* outbreak. Television was the medium that delivered most information, followed by radio and newspapers. News website nu.nl was also a relevant source of information. Finally, social media played a marginal role, whereby social network sites were more important than Twitter.

Next, we assessed what factors influence passive information consumption for each channel or source (dependent variables). Results for the different regression analyses can be found in Table 4 (each column representing the regression analysis for a specific medium). Time spent on the medium was the most influential predictor for passive consumption of information for several media or sources. Interest in health information, and perceived health influenced passive consumption of information for all media and sources, except for social media. Perceived severity of the *Salmonella* outbreak played a small role in the passive consumption of outbreak-related information through traditional media. The other factors and demographics played no or a marginal role, with one exception for age in the case of nu.nl (a popular news website in the Netherlands), where lower age was an important predictor.

**Table 4:** Predictors of passive information consumption for popular channels and sources (n = 1,057)

	**TV**	**Radio**	**Paper**	**Nu.nl**	**Newspaper website**	**Twitter**	**Facebook/Hyves**
R^2^	.17	.22	.30	.11	.08	.09	.09
	Beta	Beta	Beta	Beta	Beta	Beta	Beta
Perceived severity Salmonella			.06				
Perceived severity Salmonella outbreak	.11**	.09**	.08*			.07	
Carefulness with salmon preparation			.06	.09**			
Carefulness with general food preparation					.15***	.06	.13***
Interest in health information	.25***	.17***	.16***	.15***	.22***		.06
Perceived health	.10**	.10**	.06	.10*	.07*		
Knowledge about Salmonella						-.08*	
Time spent with/on medium	.24***	.40***	.45***	.11***	.09**	.25***	.17*** (Facebook) .11** (Hyves)
Age	-.06			-.25***	-.08*	-.10**	-.11**
Education	-.08*						-.07
Children		.06		.07*			
Sex	-.15***	-.10**	-.05	-.07	-.11**		-.06
Income	.07*		.06*				

Note: ‘Children’ assessed as 0 = no, 1 = yes; ‘Sex’ assessed as 0 = male, 1 = female; ‘Time spent on Twitter as 0 = no, 1 = yes.

*p < .05 **p < .01 ***p < .001.

We also encountered active information behavior among the respondents. Ninety-one respondents (8.6%) had a specific question about *Salmonella* infections in general or the outbreak in particular, or searched for more information. They turned to television (17 respondents), radio (8 respondents), a newspaper (20 respondents), the Internet (77 respondents), or a combination of several media. If they used the Internet, people surfed to:

•The website of the Netherlands Food and Consumer Product Safety Authority (NVWA) (22 respondents);

•The website of their Municipal Health Service (21 respondents);

•The website of a newspaper (16 respondents);

•The website of the Food Center, a Dutch authority that provides the general public with information about healthy and safe food consumption (16 respondents);

•Wikipedia (16 respondents);

•The website of the National Institute for Public Health and the Environment (RIVM) (12 respondents);

•The website of the company that was the source of the outbreak (6 respondents);

•Facebook or Hyves – a Dutch social network (3 respondents);

•A different website (15 respondents).

Finally, we focused on a specified range of online sources, and if a website was visited by a respondent, we asked how the website was found, whether it provided the information the respondent was looking for, how satisfied he/she was with it, and whether he/she trusted the information. The number of respondents who answered these questions was relatively low (ranging from 16 for the NVWA website, to 3 for Facebook and Hyves). Most online sources were either found through a Google search or directly by entering the URL. The NVWA website and Wikipedia were predominantly found through a Google search, and newspaper websites were mostly accessed directly. Virtually all sources provided the seekers with the information they were looking for. Satisfaction with the source was high for Wikipedia, the NVWA website, and the website of the Municipal Health Service, while it was low for Facebook and Hyves. Trust in the online source was relatively high for the websites of the government organizations: the RIVM, the NVWA, and the Municipal Health Service. Trust in the website of the company that was the source of the outbreak and of the social networks Facebook and Hyves was relatively low.

## Discussion

### Perceptions

Our results show that shortly after *Salmonella Thompson* broke out nationally in the Netherlands, the general public perceived *Salmonella* gastro-enteritis as a serious illness, comparably severe to asthma and diabetes. They also perceived the outbreak as severe.

### Knowledge

Respondents’ knowledge of *Salmonella* (gastro-enteritis) was appropriate, except for the common food sources of a *Salmonella* infection, the duration of the incubation period, and the fact that treatment with antibiotics is usually not needed. This study reveals gaps in the public’s knowledge on *Salmonella* infections, and shows where health education efforts should be put in by health organizations. Moreover, it also shows that it is important to assess existing public knowledge regarding different infectious diseases, in order to improve health communication, and to fill knowledge gaps.

### Preventive behavior

Despite warnings through mass media channels, the majority of the respondents neither checked whether they had contaminated batches of smoked salmon products at home, nor did their kitchen hygiene increase during the outbreak. While the perceived severity of the outbreak influenced the adoption of preventive measures to some degree, increased general kitchen hygiene during the outbreak appeared to be the most important antecedent. This suggests that being careful to avoid a food-borne infection during an outbreak is primarily done by people who are already concerned about food safety. Since salmon is very popular and processed in many other products, it is well possible that people did not realize they owned contaminated products. Some people even knowingly ate contaminated salmon, thereby neglecting health officials’ advice to throw contaminated salmon away, or to return it to the supermarket.

### Sources of information

During the infectious disease outbreak, the general public mostly receives information through traditional media and popular news(paper) websites. Health organizations should focus on these media to inform the general public, and to persuade them to take preventive actions. We came to a similar conclusion after studying information behavior during the 2011 German EHEC outbreak [13]. We uncovered that people do not use social media in these situations, as they think health-related information is ‘out of place’ there, or unreliable [13]. Investing time and effort in a social media campaign may serve only a very small portion of the population, resulting in a low return on investment.

The consumption of outbreak-related information through a traditional medium and Twitter was mostly determined by time spent on the medium, suggesting that consuming outbreak-related information is for a large part coincidental, and highly determined by the news selection of the different media. A higher interest in health information also resulted in more outbreak-related information consumption. However, this could also be due to a recall bias, as those interested in such information might more easily remember receiving it. Other predictors played no or a marginal role, with the exception of lower age for the popular Dutch news website nu.nl.

Only a small sample of our respondents actively searched for information about *Salmonella* or the outbreak. Those who did mostly turned to the Internet. There, they consulted multiple sources, found through a Google search or by entering the URL, like national food safety institutes, online newspapers, websites of Municipal Health Services, and Wikipedia. The latter has also been found to be an important source of information during other infectious disease outbreaks [13,28]. It should be noted, however, that the popularity of Wikipedia could be due to the high ranks it receives in Google. The website of the National Institute for Public Health and the Environment (the Dutch equivalent of the American Centers for Disease Control and Prevention) was consulted less than the aforementioned sources. This implies that such national institutes should not solely rely on their own communication efforts, but they should collaborate with local health organizations, and they should contribute to relevant Wikipedia articles. There has been some debate, however, concerning the quality of Wikipedia articles for the goal of public health education, and studies on this matter show mixed results. The quality of medical Wikipedia articles has been found to be good but inferior to official patient information [29], of similar quality as official patient information [30], or incomplete, which might have harmful effects [31]. These results imply that if health organizations decide to use Wikipedia to inform the public during a large-scale *Salmonella* outbreak, they should make a continuous effort to continuously monitor the relevant articles and to improve their quality.

Our analysis did not result in a clear set of predictors for consuming outbreak-related information through social media. Also, the predictors that are often found for consuming health information through traditional media (like interest in health information, and perceived health) did not hold for these services. If we are to find a set of predictors for this context – presuming they do exist, considering the little use the general public made of social media during the outbreak – we will have to step off the beaten path and gather a set of new predictors.

### Limitations

We conducted the survey at the end of the *Salmonella* outbreak. While this allows for a good retrospective view, the general public’s perceptions and behavior may evolve during an outbreak. Different phases induce different information needs, related to the uncertainties of the situation (e.g., fear may play a bigger role when the outbreak source is still unknown) [32]. A longitudinal setup would provide insight in these developments, and it would be an interesting direction for future research. Second, the number of people in our study that actively searched for more information or for answers to their questions was relatively low. It is therefore difficult to base generalizable conclusions on these results, and our efforts should be viewed as explorative. They do provide valuable input for in-depth studies aimed at assessing people’s outbreak-related information seeking processes. Such studies have already generated important insights for the health domain (e.g., [33]). But it is also possible that, in this context, people actively searching for more information is a rarity, possibly due to the fact that the information provided by the different media is perceived as adequate. Other studies should acknowledge or refute this thesis. Finally, our study was restricted to the Dutch general public. We do not have any indications that these results would not hold for other western European countries, but these should be validated for countries where the process of outbreak-related information provision and the Internet penetration rate are fundamentally different.

## Conclusion

This study aimed to determine which information health organizations should convey during a large-scale *Salmonella* outbreak, and by which channel, to maximize citizen compliance with preventive advice. We found that after the outbreak, the general public perceived *Salmonella* gastro-enteritis as severe, but the public did not wholeheartedly apply the advised preventive measures. Health organizations should use traditional media, and news and newspaper websites to inform the public, and to persuade them to take preventive actions. They should increase knowledge about *Salmonella* infections, and stimulate citizens to check for possibly contaminated products at their home, and to increase kitchen hygiene.

Future research should focus on the role Wikipedia can play during infectious disease outbreaks, not only those caused by *Salmonella*. We are especially interested in case studies in which health organizations have used Wikipedia as a public health education tool, and in how they experienced this in terms of public appreciation, and organizational investment. Furthermore, studies assessing the quality and completeness of health-related Wikipedia articles can be very valuable in helping health organizations decide on which articles they should use or improve the quality of. Finally, our study pointed out that there is a group of people who knowingly take risks by eating contaminated products during a *Salmonella* outbreak. A future study should focus on this group, and uncover their motivations for doing so (e.g., by interviewing patients with an infection who were seen by doctors during a *Salmonella* outbreak), to improve health education for this group.

## Abbreviations

EHEC: Enterohaemorrhagic E. Coli; NVWA: the Netherlands Food and Consumer Product Safety Authority; RIVM: National Institute for Public Health and the Environment.

## Competing interests

The authors declare that they have no competing interests.

## Authors’ contributions

LvV contributed to the study design and collection of data, analyzed the data, and drafted the manuscript as the lead writer. DJMAB contributed to the study design and collection of data, and critically reviewed the first draft of the paper. JEWCGP and JES contributed to the study design. AT contributed to the study design and collection of data, and critically reviewed the first draft of the paper. All authors approved the final version.

## Pre-publication history

The pre-publication history for this paper can be accessed here:

http://www.biomedcentral.com/1471-2458/14/100/prepub

## Supplementary Material

Additional file 1 Survey. Click here for file
