# Supplementary material for: Public knowledge and preventive behavior during a large-scale Salmonella outbreak: results from an online survey in the Netherlands
Source: BMC Public Health. 2014 Jan 31;14:100. doi: 10.1186/1471-2458-14-100 (PMC3913330; doi:10.1186/1471-2458-14-100)
Supplement: Additional file 1 — Survey. [file 1471-2458-14-100-S1.docx]

**Perceived severity *Salmonella***

1. When you contract a *Salmonella* infection, that’s serious.

[five-point Likert scale, ranging from 1 (totally disagree) to 5 (totally agree)]

2. A *Salmonella* infection is very damaging to my health.

[five-point Likert scale, ranging from 1 (totally disagree) to 5 (totally agree)]

3. How serious do you think it would be, if you would get one of the following diseases in the coming year?

|  | Not serious at all | Not serious | Quite serious | Serious | Very serious |
| --- | --- | --- | --- | --- | --- |
| Ordinary flu | o | o | o | o | o |
| Diabetes | o | o | o | o | o |
| Heart attack | o | o | o | o | o |
| *Salmonella* infection | o | o | o | o | o |
| Asthma | o | o | o | o | o |
| HIV or AIDS | o | o | o | o | o |

**Perceived severity *Salmonella* outbreak**

4. In the last couple of weeks, were you concerned about the *Salmonella* outbreak caused by contaminated salmon?

[five-point Likert scale, ranging from 1 (not concerned at all) to 5 (very concerned)]

5. In the last couple of weeks, how afraid were you of the *Salmonella* outbreak caused by contaminated salmon?

[five-point Likert scale, ranging from 1 (not afraid at all) to 5 (very afraid)]

6. In the last couple of weeks, how often did you think about the *Salmonella* outbreak caused by contaminated salmon?

[five-point Likert scale, ranging from 1 (never) to 5 (constantly)]

**Knowledge of *Salmonella* infections**

7. We will now give you nine statements about *Salmonella*. Please indicate whether you think the statement is correct or incorrect.

- People can contract *Salmonella* by eating contaminated food. (correct)

- *Salmonella* is present predominantly in chicken, raw vegetables, and fruit. (correct)

- After you have eaten *Salmonella*-contaminated food, it can take weeks before you become ill. (incorrect, usually within 6 to 72 hours)

- By thoroughly washing and heating food, you can prevent contracting *Salmonella*. (correct)

- Usually, people contract *Salmonella* from someone who already has it. (incorrect)

- If you are having *Salmonella*-related symptoms (e.g., vomiting, diarrhea), you are temporarily not allowed to work in healthcare. (correct)

- *Salmonella* is almost always treated with antibiotics. (incorrect)

- If there is an outbreak (i.e., a large group of people has contracted *Salmonella*), then the Municipal Health Service will try to trace the source of the outbreak. (correct)

- *Salmonella* can be very serious, especially for babies and the elderly. (correct)

**Infection in vicinity**

8. In the last couple of weeks, have you yourself contracted *Salmonella* from eating contaminated salmon?

[yes/no]

9. In the last couple of weeks, has someone in your vicinity (family or friends) contracted *Salmonella* from eating contaminated salmon?

[yes/no]

**Behavior after hearing about the *Salmonella* outbreak**

10. After hearing about the *Salmonella* outbreak, have you checked if you had contaminated salmon at home?

[yes/no]

11. What have you done with the salmon you had at home?

I threw away all salmon

The salmon I had at home was not contaminated

I had contaminated salmon, and threw it away

I had contaminated salmon, but did eat it

Something else, namely…

**Applying measures to prevent a *Salmonella* infection**

12. In the last couple of weeks, I have eaten less salmon than normally.

[five-point Likert scale, ranging from 1 (totally disagree) to 5 (totally agree); with the addition of an ‘I do not eat salmon’ option]

13. In the last couple of weeks, I have been careful with buying salmon or products with salmon as an ingredient.

[five-point Likert scale, ranging from 1 (totally disagree) to 5 (totally agree); with the addition of an ‘I do not eat salmon’ option]

14. In the last couple of weeks, I have bought canned salmon instead of fresh salmon.

[five-point Likert scale, ranging from 1 (totally disagree) to 5 (totally agree); with the addition of an ‘I do not eat salmon’ option]

**Increased kitchen hygiene**

15. In the last couple of weeks, I have washed my hands before cooking more often than normally.

[five-point Likert scale, ranging from 1 (totally disagree) to 5 (totally agree)]

16. In the last couple of weeks, when I was cooking, I was paying more attention to hygiene than normally.

[five-point Likert scale, ranging from 1 (totally disagree) to 5 (totally agree)]

17. In the last couple of weeks, I have paid extra attention to thoroughly heating meat and fish products.

[five-point Likert scale, ranging from 1 (totally disagree) to 5 (totally agree); with the addition of an ‘I do not eat meat and fish’ option]

**Interest in health information**

18. Are you interested in health information?

[five-point Likert scale, ranging from 1 (not interested at all) to 5 (very interested)]

19. How often do you read health information on the Internet, in flyers or in newspapers?

[five-point Likert scale, ranging from 1 (never) to 5 (very often)]

20. How often do you watch programs about health or healthcare on TV?

[five-point Likert scale, ranging from 1 (never) to 5 (very often)]

21. How often do you talk to others about your health or healthcare in the Netherlands?

[five-point Likert scale, ranging from 1 (never) to 5 (very often)]

**Perceived health**

22. How would you describe your health?

[five-point Likert scale, ranging from 1 (bad) to 5 (excellent)]

23. How concerned are you about your health?

[five-point Likert scale, ranging from 1 (not concerned at all) to 5 (very concerned); rescaled for analysis]

24. I am ill more often than other people of the same age and sex.

[five-point Likert scale, ranging from 1 (totally disagree) to 5 (totally agree); rescaled for analysis]

**Exposure to *Salmonella* information**

25. In the last couple of weeks, how often have you heard or read about the *Salmonella* outbreak?

|  | (almost) Never | About once a week | Once every few days | About once a day | Several times a day |
| --- | --- | --- | --- | --- | --- |
| On TV | o | o | o | o | o |
| On the radio | o | o | o | o | o |
| In the newspaper | o | o | o | o | o |
| On nu.nl | o | o | o | o | o |
| On newspaper websites | o | o | o | o | o |
| On Twitter | o | o | o | o | o |
| On Facebook or Hyves | o | o | o | o | o |

Note. Nu.nl is a popular Dutch news website; Hyves is a Dutch social network, comparable to Facebook.

**Actively searching for information**

26. In the last couple of weeks, you may have had a question about the *Salmonella* outbreak. Or you may have just wanted to know more about it. If this was the case, you may have searched for information, for example on the Internet or in your newspaper. Have you searched for information?

Yes, I have searched for information or answers to my questions

[if yes, participants were directed to question 27]

No, I have not searched for information or answers to my questions

[if no, participants were directed to question 32]

27. Where have you searched for information on the *Salmonella* outbreak?

On TV

On the radio

In the newspaper

On the Internet, namely on

Wikipedia

Facebook or Hyves

A newspaper website (e.g., volkskrant.nl)

The website of the Dutch National Institute for Public Health and the Environment (rivm.nl)

The website of the Netherlands Food and Consumer Product Safety Authority (nvwa.nl)

The website of the company that was the source of the outbreak (foppenpalingenzalm.nl)

The website of the Food Center (voedingscentrum.nl)

The website of your Municipal Health Service

A different website, namely…

[if a participant selected only the option TV, radio, or newspaper, they were directed to question 33; if they chose one of the websites, they were directed to question 28, where they were asked to answer questions 28 through 32 for only one of the websites they had consulted. This website was chosen at random]

28. You indicated that you have searched for information on [insert name of website]. Did you end up at this website through Google? Or did you go to this website directly?

I found this website through Google

I visited this website directly

29. You indicated that you have searched for information on [insert name of website]. Did you find the information you were looking for? Or did you find an answer to your question?

Yes, I found what I was looking for on this website

No, I did not find what I was looking for on this website

30. How satisfied were you with the information you found on [insert name website]?

[five-point Likert scale, ranging from 1 (not satisfied at all) to 5 (very satisfied)]

31. How much did you trust the information you found on [insert name website]?

[[five-point Likert scale, ranging from 1 (not at all trustworthy) to 5 (completely trustworthy)]

**Demographics**

32. How many hours a day…

| Do you watch TV? | I don’t have a TV | Less than 1 hour | 1 to 2 hours | 2 to 3 hours | 3 to 4 hours | More than 4 hours |
| --- | --- | --- | --- | --- | --- | --- |
| Do you listen to the radio? | I don’t have a radio | Less than 1 hour | 1 to 2 hours | 2 to 3 hours | 3 to 4 hours | More than 4 hours |
| Do you read the newspaper? | I don’t have a newspaper | Less than 1 hour | 1 to 2 hours | 2 to 3 hours | 3 to 4 hours | More than 4 hours |
| Are you on the Internet? | I don’t have access to the Internet | Less than 1 hour | 1 to 2 hours | 2 to 3 hours | 3 to 4 hours | More than 4 hours |

33. Do you have a Twitter account?

Yes

No

34. How often are you on Facebook?

I don’t have a Facebook account

(almost) Never

About once a week

Once every few days

About once a day

Several times a day

35. How often are you on Hyves?

I don’t have a Hyves account

(almost) Never

About once a week

Once every few days

About once a day

Several times a day
